# Female-bias in systemic lupus erythematosus: How much is the X chromosome to blame?

**DOI:** 10.1186/s13293-024-00650-y

**Published:** 2024-10-07

**Authors:** Adriana A. Vieira, Inês Almada-Correia, Joana Inácio, Patrícia Costa-Reis, S. T. da Rocha

**Affiliations:** 1 Rheumatology Research Unit, Instituto de Medicina Molecular João Lobo Antunes, Lisbon, Portugal; 2 Faculdade de Medicina, Universidade de Lisboa, Lisbon, Portugal; 3 Faculdade de Ciências e Tecnologia, Universidade Nova de Lisboa, Lisbon, Portugal; 4 Pediatric Rheumatology Unit, Pediatrics Department, Hospital de Santa Maria, Lisbon, Portugal; 5 iBB - Institute for Bioengineering and Biosciences, Department of Bioengineering, Instituto Superior Técnico, Universidade de Lisboa, Lisbon, Portugal; 6 Associate Laboratory i4HB Institute for Health and Bioeconomy, Instituto Superior Técnico, Universidade de Lisboa, Lisbon, Portugal

**Keywords:** Systemic lupus erythematosus, X-Chromosome inactivation, Epigenetics, Auto-immunity.

## Abstract

Systemic lupus erythematosus (SLE or lupus) is an immune-mediated disease associated with substantial medical burden. Notably, lupus exhibits a striking female bias, with women having significantly higher susceptibility compared to men, up to 14-fold higher in some ethnicities. Supernumerary X chromosome syndromes, like Klinefelter (XXY) and Triple X syndrome (XXX), also present higher SLE prevalence, whereas Turner syndrome (XO) displays lower prevalence. Taken together, SLE prevalence in different X chromosome dosage sceneries denotes a relationship between the number of X chromosomes and the risk of developing lupus. The dosage of X-linked genes, many of which play roles in the immune system, is compensated between males and females through the inactivation of one of the two X chromosomes in female cells. X-chromosome inactivation (XCI) initiates early in development with a random selection of which X chromosome to inactivate, a choice that is then epigenetically maintained in the daughter cells. This process is regulated by the *X-Inactive-Specific Transcript* (*XIST)*, encoding for a long non-coding RNA, exclusively expressed from the inactive X chromosome (Xi). XIST interacts with various RNA binding proteins and chromatin modifiers to form a ribonucleoprotein (RNP) complex responsible for the transcriptional silencing and heterochromatinization of the Xi. This ensures stable silencing of most genes on the X chromosome, with only a few genes able to escape this process. Recent findings suggest that the molecular components involved in XCI, or their dysregulation, contribute to the pathogenesis of lupus. Indeed, nonrandom XCI, elevated gene escape from XCI, and the autoimmune potential of the XIST RNP complex have been suggested to contribute to auto-immune diseases, such as lupus. This review examines these current hypotheses concerning how this dosage compensation mechanism might impact the development of lupus, shedding light on potential mechanisms underlying the pathogenesis of the disease.

## Abstract (plain language summary)

Lupus is a disease where the immune system mistakenly attacks the body’s own tissues, leading to a range of complicated health issues. Interestingly, lupus is much more common in women (XX) than in men (XY), with women being up to 14 times more likely to develop the condition. Additionally, some genetic conditions involving extra or missing X chromosomes can also affect the chances of getting lupus: Klinefelter (XXY) and Triple X (XXX) syndromes show higher rates of lupus, while conditions like Turner syndrome (XO) have a lower risk. This suggests a link between the number of X chromosomes and the likelihood of developing the disease.

In female cells, a process called X-chromosome inactivation (XCI) occurs, where one of the two X chromosomes in each cell is switched off to equalize X chromosome dosage with males. This process is regulated by a gene called *XIST*, which produces a long non-coding RNA. XIST helps to form a complex of RNA and proteins that silence the inactive X chromosome (Xi), ensuring stable gene expression patterns.

Recent research suggests that molecular components or problems with this process might be linked to lupus. This review focuses on three hypotheses in which XCI or its dysregulation could impact lupus: nonrandom XCI, incomplete silencing of certain genes on the Xi, and the potential for the XIST ribonucleoprotein complex to activate the immune system. By investigating these mechanisms, researchers aim to better understand how variations in XCI mechanisms contribute to the development of lupus.

## Highlights

Lupus is an immune-mediated disease with higher prevalence in women than men.

Individuals with supernumerary X chromosomes have a higher lupus susceptibility and there is lower prevalence in individuals with Turner syndrome (X0), suggesting that X chromosome dosage may be implicated in lupus pathogenesis.

The X chromosome harbors several genes involved in immune responses, some of which are implicated in lupus pathogenesis.

The X-linked content between female and male genomes is balanced by a dosage compensation mechanism regulated by the XIST non-coding RNA, known as X-chromosome inactivation (XCI), which silences one of the two X chromosomes in female cells.

The molecular components involved in XCI or their dysregulation may actively contribute to lupus pathogenesis in several ways: 1) through the preferential inactivation of one X chromosome (XCI skewing); 2) by increasing XCI escape of X-linked genes involved in immune responses, such as *TLR7*, *CXCR3*, and *CXorf21*; 3) by being preferential targets for autoimmune responses, as it might be the case for XIST and its protein interactors.

## Introduction

Systemic lupus erythematosus (SLE or Lupus; OMIM 152700) is an immune-mediated disease associated with a lower life expectancy due to an increased risk of major infections and renal and cardiovascular complications [1, 2] Like other rheumatic diseases, such as Sjögren’s syndrome, systemic sclerosis, rheumatoid arthritis and multiple sclerosis, lupus affects women more frequently than men. The female-to-male ratio in lupus is 4–14:1, depending on the ethnicity [3]. The medical burden is significant as lupus remains one of the leading causes of non-traumatic death in young women [4]. This review delves into the factors contributing to the elevated susceptibility of females to develop lupus. Specifically, we investigate the influence and potential causality of the components of X-linked dosage compensation process and its dysregulation in the initiation and progression of lupus.

## Lupus overview

**Fig. 1:**
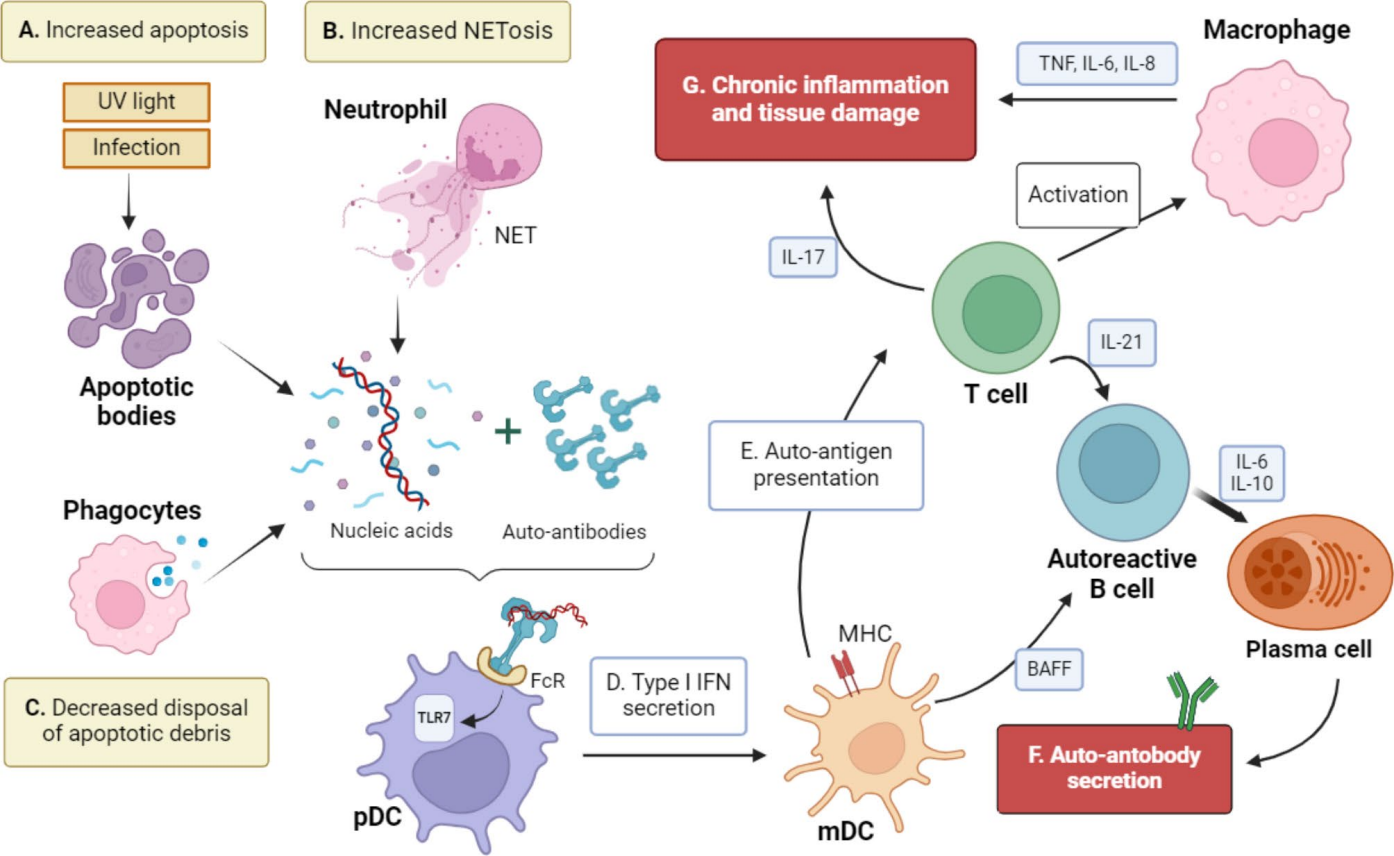
Lupus pathogenesis. Legend: Increase in apoptosis (**A**) and in NETosis (**B**) and decreased capacity to dispose apoptotic residues (**C**) leads to increased auto-antigen exposure. These auto-antigens bind to stochastically generated autoantibodies, forming immune-complexes. Plasmacytoid dendritic cells (pDC) recognize the immune complexes, activating toll-like receptors (e.g. TLR7) and increasing type I interferon (IFN) response (**D**). Auto-antigen presentation to T cells results in a higher production of IL-17 and a pro-inflammatory response (**E**). A higher production of B cell activation factor (BAFF) leads to a prolonged survival of autoreactive B cells, which secrete interleukin (IL)-6 and 10, turn into plasma cells and produce auto-antibodies (**F**). Macrophages are activated, producing TNF, IL-6 and IL-8. This vicious cycle ultimately results in chronic inflammation and tissue damage (**G**). Created with BioRender.com

In SLE there is an imbalance between the production and the disposal of apoptotic material (Fig. 1). SLE patients have an increase in apoptosis, which is further intensified by external factors, such as UV light and infections, well-known SLE triggers (Fig. 1A). NETosis, a regulated form of cell death by neutrophils that entails the formation of neutrophil extracellular traps (NETs), also contributes to SLE pathogenesis (Fig. 1B). In patients with SLE, neutrophils are more prone to form NETs, and there is an accumulation of these traps, formed by nucleic acids-protein complexes [5, 6], which are another source of auto-antigens. In addition, SLE patients have defective mechanisms of apoptotic waste removal and clearance of NETs, which globally cause auto-antigen exposure [6] (Fig. 1C).

These autoantigens bind to stochastically generated autoantibodies and activate plasmacytoid dendritic cells (pDCs). These cells produce high amounts of interferon (IFN) α upon immune complexes uptake and toll-like receptors (TLRs) activation, thereby contributing to the characteristic IFN-α signature described in lupus (Fig. 1D). IFN-α promotes the activation of B and T lymphocytes, enhancing the overall effect.

The auto-antigens are also presented by dendritic cells to stochastically generated autoreactive B cells in the germinal centers of secondary lymphoid organs (Fig. 1E). Once activated, B cells expand, mature, and secrete auto-antibodies against nuclear antigens, a unifying feature of lupus (Fig. 1F). These auto-antibodies are high affinity, somatically mutated IgG, which suggests that they have arisen in the germinal center, where T and B lymphocytes interact to promote class switching. The immune complexes activate complement and induce inflammation, causing tissue damage. Other cytokines also participate in this amplification loop, including BAFF (B cell activation factor), interleukin (IL)-6, IL-10, IL-17 and IL-23. IL-2 is decreased in lupus and contributes to the imbalance between Th17 cells and regulatory T cells (Treg) (Fig. 1G). SLE pathogenesis is, therefore, a vicious cycle of auto-antigen exposure, auto-antibody production, chronic inflammation, and tissue damage.

SLE murine models have been helpful in unveiling lupus pathogenesis [7]. There are several types of SLE mouse models, including spontaneous models, like the NZB/NZW F1, NZM2328 or MRL/Ipr mice, which develop lupus-like symptoms naturally, and induced models, such as the pristane-induced lupus model, where the disease is triggered by this naturally occurring saturated hydrocarbon. These models help researchers study various aspects of SLE pathogenesis and potential treatments. However, while mouse models have been instrumental in advancing our understanding of lupus, accurately replicating the human characteristics of the disease remains challenging due to its heterogeneity and the complex interplay of genetic, epigenetic, and environmental factors [8]. The short lifespan of mice further complicates the development of models that represent the long-term progression and chronicity of human SLE [9]. Despite their significance in studying pathogenic mechanisms, caution is needed when translating findings from mice to humans due to inherent differences. Addressing the sex bias in lupus and other immune-mediated diseases requires a multifaceted strategy that integrates patient-derived data with animal models and fosters collaboration among geneticists, immunologists, and molecular experimentalists [8].

### Female-bias in lupus

Women are, generally, less susceptible to infectious diseases than men (reviewed in [10–12]). A side effect of a more robust immunological response to infections is a higher predisposition to autoimmunity [13, 14]. Lupus stands as a paradigmatic example of a disease characterized by a marked bias toward women, with a female-to-male ratio of up to 14:1 [3].

Female sex hormones, specifically estrogen, are believed to play a role in the female predisposition for immune-mediated diseases, with early menarche associated with increased risk of lupus [15, 16]. Estrogens are known to have immunostimulatory effects, regulating TLR-dependent responses of dendritic cells through the estrogen nuclear receptor Erα (reviewed in [17]). Furthermore, estrogen can increase antigen presentation, pro-inflammatory cytokines levels, and the production of double stranded-DNA antibodies [16, 18].

Nevertheless, the female sex bias found in lupus cannot be solely attributed to hormone exposure, as it persists both before puberty and after menopause [19, 20]. In 2008, a transgenic mice model offered the initial evidence supporting that besides female hormones, the number of X chromosomes also contributes to the female bias observed in lupus. In this study, the authors generated both transgenic ovarian- and testes-bearing XX and XY mice. They observed that both transgenic ovarian- and testes-bearing XX mice had greater susceptibility to lupus compared to transgenic ovarian- and testes-bearing XY mice upon treatment with pristane, a naturally occurring saturated hydrocarbon commonly used as an inducer of a lupus-like disease in rodents. Thus, chromosomal content (XX vs. XY) and not the gonadal types (ovarian vs. testes) segregated with disease susceptibility in this murine model of lupus [21]. These findings were also reproduced in a spontaneous NZM2328 mouse model of lupus [22].

**Fig. 2:**
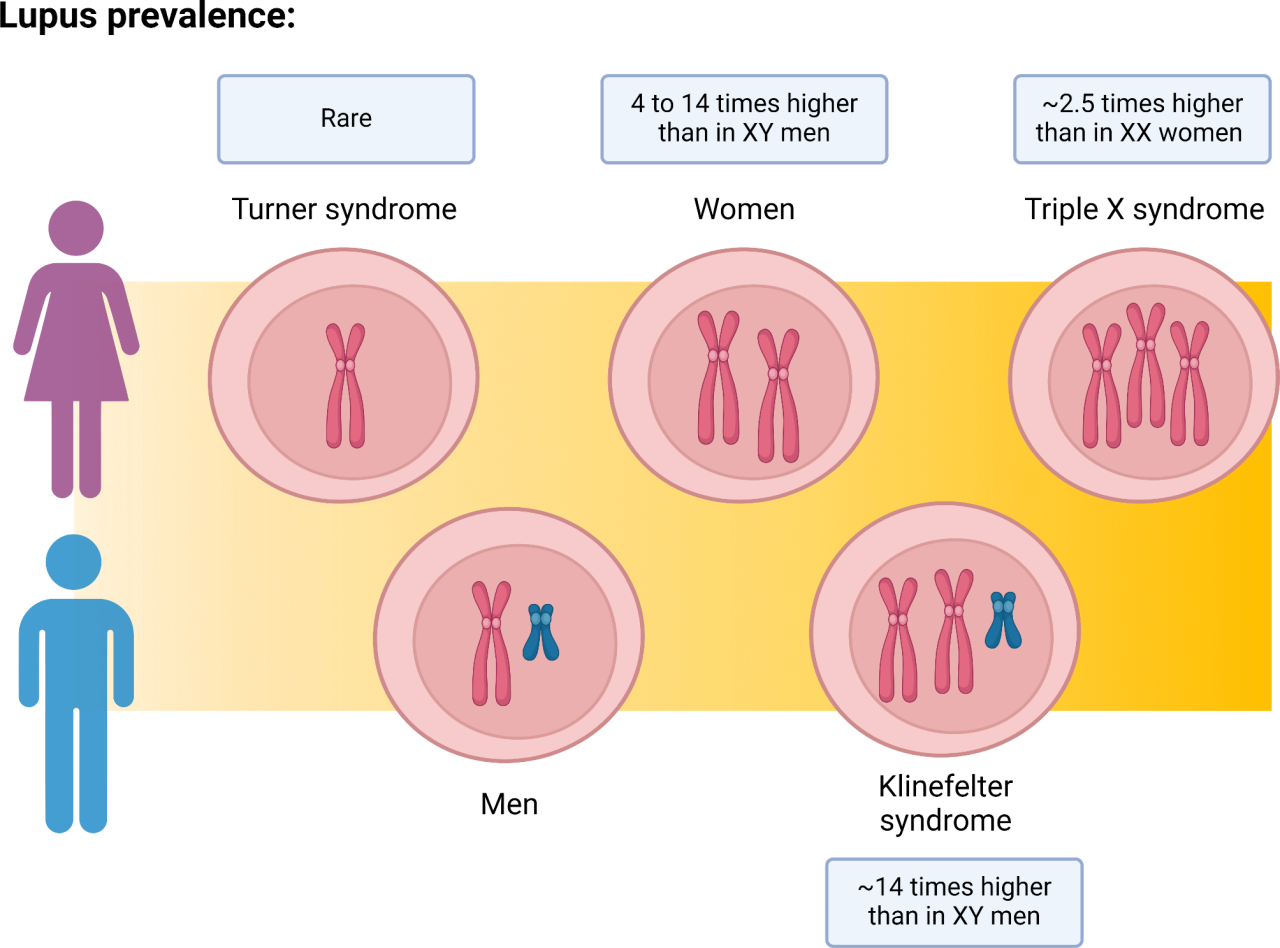
The number of X chromosomes is a risk factor for developing lupus. Legend: Lupus presents a marked sex-bias with females with unaltered karyotypes (46, XX) having 4 to 14 times higher prevalence than males with typical karyotypes (46, XY) [3]. In sex chromosome aneuploidies, there is a clear spectrum of SLE predisposition according to the number of X chromosomes: cases of concurrent SLE and Turner syndrome (45, XO) are rare [24], whereas supernumerary X chromosomes syndromes, like Klinefelter (47, XXY) and Triple X syndrome (47, XXX), display a higher SLE prevalence than normal karyotyped individuals [23, 25]. Pink chromosome: X-chromosome; Blue chromosome: Y chromosome. Created with BioRender.com

Further evidence for a link between the X chromosome and SLE predisposition came from studies looking at individuals with an atypical number of X chromosomes (Fig. 2). For instance, women with triple X syndrome (47, XXX) were shown to have a ~ 2.5 higher SLE prevalence than women with an unaltered karyotype [23]. Conversely, women with Turner syndrome (45, X0) are known to present a lower risk of developing lupus [24]. In addition, men with Klinefelter syndrome (47, XXY) exhibit a SLE risk similar to that of women, and it is approximately 14 times greater than that of men with a typical karyotype [25]. These discoveries suggest that susceptibility to lupus may involve a gene dosage effect of the X chromosomes, with a few X-linked immune-related genes potentially implicated.

The X chromosome harbors several genes involved in immune responses. Among them, there are genes encoding for toll-like receptors (*TLR7* and *TLR8*) and their signaling pathways (*CXorf21*, *IRAK1*), cytokine receptors (*CXCR3*, *IL-2RG*, *IL-3RA*, *IL-9R*), and genes involved in T-cell and B-cell activity (*BTK*, *CD40L*, *FOXP3*) (Table 1). Several of these X-linked genes have been implicated in autoimmunity and SLE pathogenesis.

**Table 1:** Title: X-linked genes with immune functions that can escape XCI in humans

Gene	Localization	Function	Cell types with XCI escape
*RPS6KA3* (ribosomal protein S6 kinase A3)	Xp22.12	Encodes a protein kinase involved in cellular signaling pathways regulating cell growth, differentiation, and response to stress.	Escapes in human pDCs from healthy women [106].
*TLR7* (Toll-Like receptor 7)	Xp22.2	Recognize single-stranded RNA from viruses and trigger immune responses.	Escapes in immortalized B cell lines [46], in B cells, monocytes, and pDCs from healthy women and Klinefelter syndrome males (XXY) [105], in pDCs and CD4 + T cells from healthy women [106, 108].
*TLR8* (Toll-Like receptor 8)	Xp22.2		Escapes in monocytes and CD4 + T cells from healthy women and Klinefelter syndrome males (XXY) [108]
*CXorf21/TASL* (Chromosome X Open Reading Frame 21, also known as *TASL*)	Xp21.2	Regulation of toll-like receptor signaling pathway.	Variable escape in primary fibroblast cell lines [118].
*CYBB* (cytochrome *b*-245 beta chain)	Xp21.1-p11.4	Encodes a protein crucial for the immune system’s ability to generate reactive oxygen species.	Escapes in human pDCs from healthy women [106].
*FOXP3* (Forkhead box P3)	Xp11.23	Development and function of regulatory T cells.	Escapes in skin fibroblasts from healthy women [119].
*IL-2RG* (Interleukin 2 receptor subunit gamma)	Xq13.1	Component of various cytokine receptors, including the IL-2 receptor, essential for immune system regulation and T cell development.	Escapes in B cells from healthy women [113].
*CXCR3* (C-X-C motif chemokine receptor 3)	Xq13.1	Role in the migration of immune cells to inflammatory sites in response to specific chemokines.	Escapes in activated T cells and immortalized B cell lines generated from pediatric lupus patients and healthy women [46].
*BTK* (Burton tyrosine kinase)	Xq22.1	Regulation of B cell development and function; a key signaling molecule in the B cell receptor pathway.	Escapes in pDCs from healthy women [106].
*IL-3RA* (Interleukin 3 receptor α chain)	Xp22.33 and Yq11.3	Contributes to the responsiveness of hematopoietic cells to IL-3 signaling.	Escapes in skin fibroblasts from healthy women [113].
*IL13RA1* (interleukin 13 receptor subunit alpha 1)	Xq24	Encodes a receptor protein involved in mediating the effects of interleukin (IL)-13, a cytokine crucial in regulating immune responses and inflammation.	Escapes in human pDCs from healthy women [106].
*CD40LG* (Cluster of Differentiation 40 Ligand)	Xq26.3	Interacts with its receptor, CD40.	Escapes in activated T cells and immortalized B cell lines generated from pediatric lupus patients and healthy women [46].
*IRAK1* (Receptor-associated kinase 1)	Xq28	Innate immune signaling and inflammatory responses.	Variable escape in primary fibroblast cell lines from healthy women [118].
*IL-9R* (Interleukin 9 receptor)	Xq28 and Yq12	Mediates the effects of IL-9, a cytokine that regulates various immune responses, including the activation and proliferation of T cells.	Escapes in skin fibroblasts derived from healthy men and women [119].

*TLR7* is the most well-studied X-linked gene associated with lupus. This gene encodes a receptor expressed in the intracellular compartments of immune cells, such as B lymphocytes, dendritic cells, and macrophages. TLR7 recognizes viral motifs in single-stranded RNA (ssRNA), particularly guanosine- and uridine-rich sequences, and triggers a signaling cascade crucial to initiate the innate immune response against viral infections. *TLR7* gain-of-function or overexpression leads to a hyperactive or promiscuous TLR7 activity, resulting in the recognition of self ssRNA and autoimmune phenotypes [26, 27]. Indeed, *TLR7* gene variants associated with gene overexpression [28] or gain-of-function have been identified in SLE patients [27, 29, 30] and TLR7 overexpression is a well-known feature of B lymphocytes and pDCs of SLE patients [31–33]. One of the gain-of-function variants, *TLR7*^Y264H,^ was sufficient to develop a lupus-like phenotype when introduced into mice [27], phenocopying the effect already seen in humans. The mutant mice presented decreased survival, increased serum levels of IFN-γ, IL-6, IL-10, and TNF, and organ involvement. This *TLR7* gain-of-function variant enhanced TLR7 activation by selectively increasing the sensing of guanosine. The overactive immune response resulted in aberrant survival of B cells and accumulation of CD11c + age-associated B cells and germinal center B cells, suggestive of an abnormal immune response [27].

In pristane-treated mice, for instance, TLR7 was specifically required for the production of RNA-reactive auto-antibodies and for the development of glomerulonephritis [34]. Moreover, in MRL lpr mice, a TLR7 antagonist prevented kidney injury, while pharmacologic activation of TLR7 induced IFN-α release, renal immune complex deposition, and renal tissue injury [35]. In addition, a *Tlr7* translocation, which caused *Tlr7* overexpression, accelerated systemic autoimmunity in a SLE mouse model [26]. Whereas a modest increase in *Tlr7* gene dosage promoted autoreactive lymphocytes with RNA specificities and myeloid cell proliferation, a substantial increase in *Tlr7* expression caused a dramatic dendritic cell dysregulation and fatal acute inflammatory injury [35]. Taken together, these findings place TLR7 at the forefront of SLE pathogenesis in connection with its presence on the X chromosome.

Downstream of the activation of TLR7 or other Toll-like receptors such as TLR8/9, it is another X-linked gene, *CXorf21*, associated with lupus. *CXorf21* also known as “TLR adaptor interacting with the SLC15A4 on the lysosome” (TASL), plays a pivotal role in the IRF5 pathway, facilitating the production of type I interferons (IFN-I) [36]. Genetic variations in *CXorf21* associated with lupus have been found in European women [37] and *CXorf21* mRNA levels in circulating blood cells serve as a biomarker of flares [38, 39]. *CXorf21* encodes a small protein of ~ 34 kDa (CXORF21) that regulates lysosomal pH, which is important for antigen processing and is expressed, preferentially, in monocytes, neutrophils, and B cells [38, 40]. Upon an immunological challenge (LPS or IFN stimulation), there is an upregulation of *CXorf21* with a greater increase in women’s monocytes compared to men’s [38]. Interestingly, CXorf21 shows dimorphic expression and a functional interaction with TLR7. *CXorf21* is overexpressed in female monocytes when compared with male monocytes at the mRNA and protein level [40, 41]. This dimorphic expression was also associated with female-biased autoimmune diseases, lupus and Sjogren’s syndrome. *CXorf21* transcript levels were increased in lymphoblastoid cell lines derived from SLE patients (Triple X females, Klinefelter syndrome males, and females and males with unaltered karyotypes), when compared with those derived from healthy males [40]. Additionally, both X-linked products, CXorf21 and TLR7, were found to co-localize at the protein level in B cells from healthy females [38].

*CD40LG* is another X-linked gene aberrantly overexpressed in T cells and occasionally in B cells from females with lupus [42–46]. This gene encodes CD40LG (also known as CD154), a surface protein primarily expressed by activated CD4 + T cells. CD40LG binds to CD40, found on various antigen-presenting cells, triggering proinflammatory responses in B cells and dendritic cells, for instance. CD40LG engagement in B cells initiates critical processes including immunoglobulin class switching and the development of memory B cells and plasma cells. In DCs, CD40LG interaction leads to heightened expression of costimulatory molecules and facilitates antigen cross-presentation, thereby bolstering T cell responses [47]. Therefore, alterations in CD40LG dosage may have an important role in SLE pathogenesis.

*CXCR3* is another X-linked gene with female-biased overexpression in CD4 + T cells of SLE patients [45]. Importantly, CXCR3 + CD4 + T cells are enriched in the kidneys and urine of patients with active lupus nephritis [48]. CXCR3 protein, also known as CD183, is a chemokine receptor that plays a critical role in directing the migration of immune cells, particularly T cells, where it is primarily expressed, to sites of inflammation or injury [49]. As such, CXCR3 has the ability to enhance autoimmunity in specific organs in diseases such as lupus.

These are just a few examples of genes located in the X chromosome that have been consistently associated with SLE pathogenesis and that might contribute to SLE sex bias.

## X-chromosome inactivation (XCI)

The sex chromosomes display a significant imbalance in gene content. On the one hand, the Y chromosome is one of the smallest in the human genome (~ 60 Mb), bearing little over 100 protein-coding genes specialized in human sex determination and in male germ cell development and maintenance [50]. On the other hand, the X chromosome is 154 Mb long and encodes more than 1000 genes [51], including several involved in immunity [52] (Table 1). Little over 50 genes on the X chromosome have functional homologues on the Y chromosome at the pseudoautosomal regions (PAR1 and PAR2), and the remaining X-linked genes are present in haploidy in the male and diploidy in the female genome [53].

**Fig. 3:**
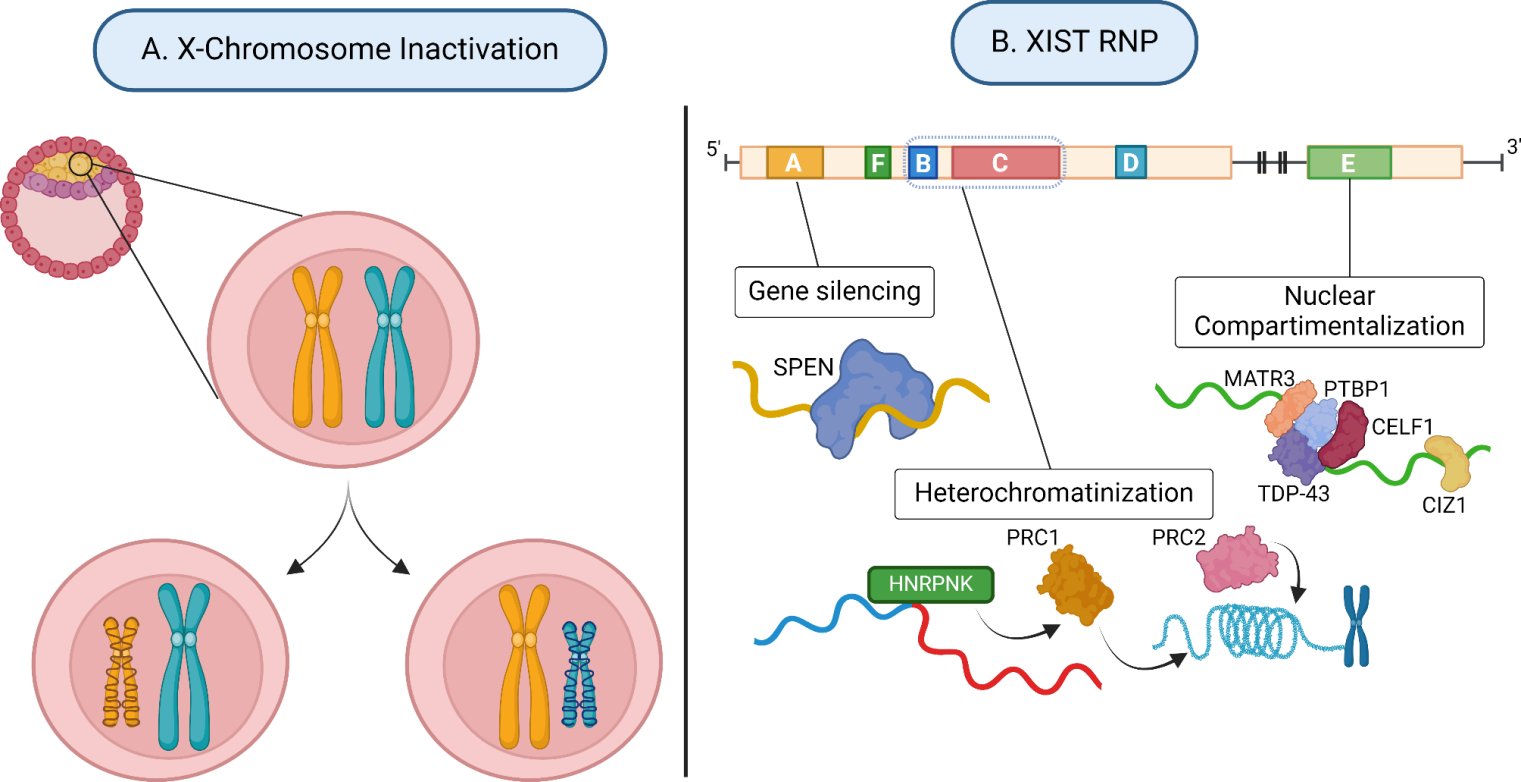
X-Chromosome Inactivation (XCI). Legend: (**A**) During embryonic development, each female cell inactivates either the maternally inherited or the paternally inherited X chromosome. From then on, each cell holds the same inactive X chromosome (Xi) and the same active X chromosome (Xa), which are stably inherited along with its epigenetic marks, as cells divide. (**B**) The XCI process is regulated by the long non-coding RNA, XIST, which is expressed *in cis* from the Xi. XIST is a ∼17-to-19 kb RNA with functional tandem repeat sequences, known as Repeats A-to-F. Repeat A recruits SPEN RNA Binding Protein (RBP) and associated repressive factors crucial for gene silencing [62]. Repeat B/C interacts with HNRPNK RBP recruiting Polycomb Group complexes (PRC1 and PRC2) responsible for heterochromatinization on the Xi [63–65] Repeat E interacts with several proteins, such as CIZ1, PTBP1, CELF1, MATR3 and TDP-43, to anchor XIST to the Xi territory and form a specialized sub-nuclear compartment crucial for XCI maintenance [66–69]. Created with BioRender.com

In mammals, the disparity in X-linked content between female and male genomes is balanced at the transcriptional level by a dosage compensation mechanism known as X-chromosome inactivation (XCI; Fig. 3A). This process involves the transcriptional silencing of one of the two X chromosomes in females to achieve gene dosage parity for X-linked genes with males [54, 55].

Early studies showed that lack of dosage compensation leads to female-specific mortality in mice [56, 57] revealing how crucial this evolutionary mechanism is for female survival in mammalian species. XCI occurs during early development, where one X chromosome in each cell of a female embryo is randomly silenced. Also, in cells with supernumerary X chromosomes (XXY, XXX, etc.), all X chromosomes except one undergo transcriptional silencing irrespectively of the biological sex [58]. This ensures the maintenance of proper gene expression of X-linked genes and prevents the potentially harmful effects of gene overdosage of multiple active X chromosomes.

In the female cells (XX) of the inner cell mass of the early blastocyst stage (~ E5 - embryonic day 5 or 5 days after fertilization in humans), both the maternal (Xm) and paternal (Xp) X chromosomes are active. The XCI process starts around implantation of the blastocyst into the mother’s uterus (from ~ E6-E10), where one X chromosome is randomly silenced and forms a heterochromatic inactive X-chromosome (Xi), classically referred as the Barr body (reviewed in [59]). Thereafter, there is an active X (Xa) and Xi chromosome within each cell (Fig. 3A). As XCI is random, the proportion of cells with active Xm or active Xp is usually 50:50. This randomness ensures an equal distribution of polymorphic X-linked gene products between the cells of a woman’s body. Once established, XCI is stably maintained as cells replicate. Daughter cells inherit the same Xi, along with its epigenetic marks, and this is faithfully maintained across all lifespans (reviewed in [59]).

The key actor of XCI is the XIST lncRNA. This gene produces a ∼17-to-19 kb spliced and polyadenylated RNA that coats the X chromosome *in cis* and promotes a cascade of events ultimately resulting in chromosome-wide transcriptional silencing, DNA methylation at gene promoters and major chromatin reorganization and condensation (Fig. 3B; reviewed in [60]). To accomplish that, XIST recruits a number of RNA Binding Proteins (RBPs) and chromatin modifiers through interaction with functional tandem repeat RNA sequences, known as Repeats A-to-F [61]. The Repeat A, the most conserved module located at the start of the XIST transcript, is crucial for transcriptional silencing by recruiting SPEN RBP and associated repressive factors to X-linked genes at the onset of XCI [62]. Repeat B/C play a pivotal role in recruiting Polycomb Group complexes through the interaction of HNRPNK RBP, being responsible for heterochromatin formation on the Xi, essential for long-lasting transcriptional silencing [63–65]. Repeat E interacts with several proteins, such as CIZ1, PTBP1, CELF1, MATR3 and TDP-43, to anchor XIST to the Xi territory and form a specialized sub-nuclear compartment crucial for XCI maintenance [66–69]. Other less well studied RNA modules, such as Repeat F and D, might also execute specialized roles. Hence, XIST RNA functions as a multifaceted molecule with distinct functional modules that work synergistically and coordinatively, overseeing the intricate process of XCI from initiation to completion.

Following the establishment of a transcriptionally silent state, the epigenetic maintenance of XCI persists through subsequent cell divisions. The epigenetic mechanisms triggered by the coating of XIST, such as DNA methylation at gene promoters, are thought to play a crucial role in the maintenance process. Early investigations indicated that XIST was not essential for silencing X-linked genes in somatic cells, as evidenced by studies demonstrating the persistence of a silent and heterochromatic Xi even after the deletion of Xist [70–73]. However, recent studies using more sensitive omics assays revealed the importance of XIST in maintaining the silent state across various somatic cell types in mice and humans (reviewed in [74]). Studies in mouse somatic cells indicated that *Xist* loss led to significant transcriptional reactivation of certain X-linked genes, with enhanced reactivation observed upon the disruption of key regulatory pathways, such as DNA methylation inhibition [75–78]. Environmental signals, including exposure to carcinogenic and inflammatory agents, further heightened the impact of losing Xist in reactivating the Xi as observed in a conditional *Xist* Knock-Out (KO) mouse model [79]. These discoveries highlight the crucial role of maintaining the silent state of the Xi for female health. Inadequate maintenance of XCI may contribute to various human diseases, particularly those exhibiting a distinct female bias, such as autoimmune diseases in general and lupus in particular.

While murine models have been instrumental in elucidating the core molecular pathways of XCI, substantial species-specific divergences between mice and humans have also been noted across the years. These differences are important for interpreting research findings and translating them into clinical applications. While the gene content between mouse and human is 95% identical, the X chromosome differs structurally. The human X chromosome is submetacentric, with a centromere dividing it into two arms, Xp and Xq. In contrast, the mouse X chromosome is telocentric, with a single arm and the centromere near one end. These structural differences may affect XCI spreading and explain why more genes evade XCI in human cells (see below).

The developmental dynamics of XCI regulation also differ significantly between mice and humans. In mice, XCI occurs in two steps: imprinted XCI silences the paternal X chromosome during pre-implantation, which is then reactivated in mid-blastocyst, followed by random XCI [80, 81]. In humans, XCI is not imprinted; instead, both X chromosomes undergo gene expression reduction (X-chromosome dampening) at the blastocyst stage, before random XCI is established [82].

The regulation of the *XIST* gene, central to XCI, also varies. In mice, *Tsix*, an antisense RNA to *Xist*, prevents *Xist* expression from the active X chromosome [82]. In contrast, in human cells, *TSIX* is co-expressed with *XIST* and has not been shown to regulate *XIST* expression the same as in the mouse [83, 84]. This may explain why XIST is expressed from both X chromosomes during X-chromosome dampening in the human blastocyst. *XIST* gene expresses a non-coding RNA that remains bound to the inactive X chromosome (Xi) in a process coined XIST coating. While Xist coating is uniform along the single arm of the murine Xi, XIST in humans preferentially covers large portions of both X chromosome arms, coinciding with H3K27me3 repressive histone mark like the mouse Xi, while regions without XIST are enriched with H3K9me3 [85].

Additionally, many more X-linked genes escape inactivation in humans (15–25%) than in mice (3–7%) [86]. The reasons for this difference are unclear, but the structure of the X chromosome may play a role, as many escape genes in humans are located on the short arm, away from the *XIST* locus on the long arm. This escape-prone X chromosome in humans allows for greater flexibility in X-linked gene expression than in mice, potentially impacting disease manifestation in different ways in the two organisms. Importantly, escape genes can evade XCI permanently (constitutive escapees) or variably (variable escapees) depending on the cell type, developmental timing, or individuals. Escapees may play crucial roles in cellular functions and can contribute to either sex- or individual-specific differences in disease susceptibility [87, 88].

### The X-chromosome inactivation and lupus

**Fig. 4:**
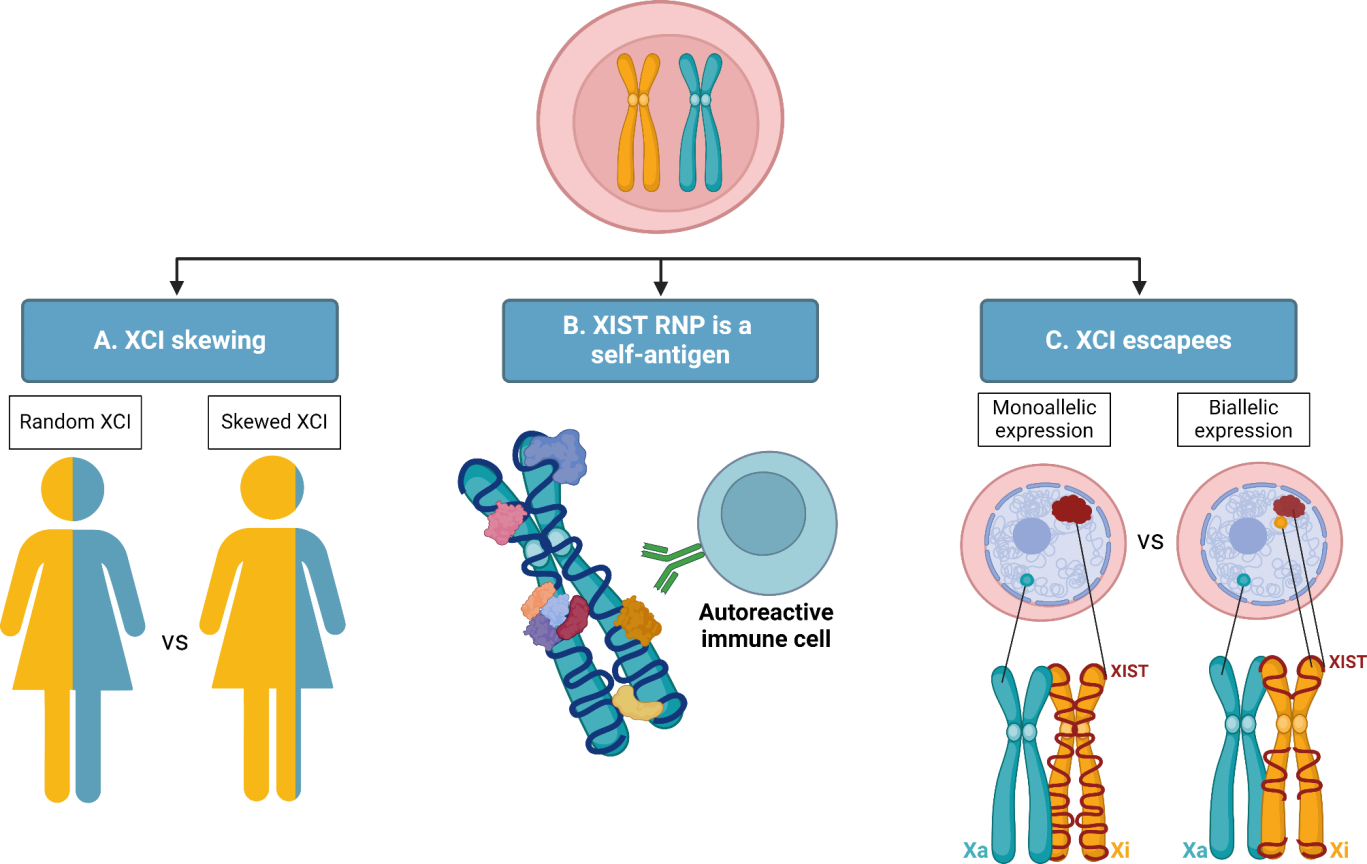
XCI dysregulation in lupus. Legend: Three hypotheses are proposed: (**A**) XCI skewing, the preferentially inactivation of one X chromosome, might impair the self-tolerance processes. (**B**) XIST RNA and its interaction with other proteins result in a ribonucleoprotein (RNP) complex that acts as auto-antigens. (**C**) Increased XCI escape can lead to biallelic expression of immune-related genes leading to a hyperactive immune response. Xi: inactive X chromosome; Xa: active X chromosome. Created with BioRender.com

The association between the number of X chromosomes and autoimmune diseases, such as lupus, has prompted scientists to explore whether the dysregulation of XCI actively contributes to the pathogenesis of these diseases. One hypothesis suggests that XCI skewing (the preferential inactivation of one of the two X chromosomes) may play a role in lupus. Another perspective considers XIST RNA and its protein interactors as potential targets for autoimmune responses, providing insight into the increased SLE propensity in individuals with extra XIST molecules, such as XXX women. In this case, it’s not XCI dysregulation itself but the presence of the factors regulating XCI that might trigger lupus. Lastly, increased escape from the Xi in women with lupus could result in aberrant overexpression of immune-related X-linked genes, like *TLR7*, influencing SLE onset and/or progression. The following discussion will delve into these possibilities in the context of SLE pathogenesis (Fig. 4).

### XCI skewing

In females, XCI induces a cellular mosaic state, characterized by patterns that vary from random (approximately 50:50), when both X chromosomes undergo equal silencing, to non-random (skewed; e.g., 80:20), indicating preferential inactivation of one X chromosome (Fig. 4A) Despite the average XCI ratio in the general female population being 50:50, skewed XCI can be observed in both women with X-linked diseases and those who are healthy [89, 90].

XCI skewing can occur through primary mechanisms, stemming from stochastic bias during early development in choosing which X chromosome to inactivate, or secondary/selective mechanisms, where specific cell types benefit from a particular XCI pattern. For instance, inactivating the X chromosome carrying a deleterious mutation can lead to clonal expansion of a certain lineage [91].

An established correlation exists between XCI skewing and aging, regardless of aging biomarkers like telomere length shortening [92–94]. Also, skewed XCI has been associated with several immune-mediated disorders, including autoimmune thyroid diseases [95] and systemic sclerosis [96]. Whether the same occurs in lupus is still a matter of debate, and does not seem necessarily a feature of other female bias immune-mediated diseases [97]. Interestingly, an RNA sequencing (RNAseq) study in B and T lymphocytes from a small cohort of SLE and healthy individuals observed a biased allelic expression profile on the X chromosome in SLE lymphocytes which may be compatible with XCI skewing [98]. To substantiate the hypothetical link between XCI skewing and lupus, further investigations with larger cohorts will be required.

How XCI skewing could lead to lupus is unclear. One hypothesis is that disturbances in the expected cellular mosaic pattern lead to a breakdown of tolerance mechanisms. In a scenario where females have random XCI, they possess two dendritic cell populations expressing either maternal or paternal X-linked self-antigens available for thymic negative selection. Potentially autoreactive thymocytes undergo negative selection by both DC populations as they pass through the thymus. However, if a female exhibits a skewed XCI pattern, such as preferential inactivation of the maternal X chromosome, in a 80:20 ratio or more, T cells will only be tolerized by DCs expressing paternal X-linked self-antigens. Consequently, autoreactive T cells specific to maternal X chromosome self-antigens may evade negative selection and enter in the circulation and cause auto-immune responses [97]. Further studies will be needed to understand whether this is indeed the case for lupus.

### XIST *RNP complex as a potential autouimmune target in lupus*

XIST lncRNA is exclusively expressed in women and men with extra X chromosomes. This RNA forms a ribonucleoprotein (RNP) complex composed by specific RNA-binding proteins (RBPs) and chromatin modifiers, which are important to establish XCI. Interestingly, many components of the XIST RNP complex have been recognized as potential targets of auto-antibodies, including SSB, also known as lupus autoantigen La [99–101]. Although these proteins are neither female-specific nor exclusively involved in XCI, the XIST RNP complex could make them more immunogenic by clustering and triggering immunoreceptor activation when exposed to the extracellular space. This idea was, recently, tested in a pristane-induced SLE mouse model by inducible transgenic expression of a non-silencing form of *Xist* in male mice. A transgenic mouse was developed with a truncation form of Xist that removes the A-repeat element required for the gene silencing of Xist, but it does not prevent the Xist RNP formation or the chromosome coating. The expression of this non-silencing form of Xist resulted in the production of autoantibodies and increased atypical B cell activity [101]. Moreover, it resulted in greater incidence and severity of glomerulonephritis, hepatic lipogranulomas, pulmonary hemorrhage and lymphohistiocytic alveolitis, reflecting disease damage in the kidney, liver, and lungs, which are often observed in severe SLE patients. While this is the first study to show that the expression of Xist in male mice is sufficient to increase SLE-like disease severity, this effect was variable and occurs only in an autoimmune-permissive (SJL/J) and not in the resistant C57BL/6J genetic background. A larger number of animals and the use of additional murine lupus models would be needed to strengthen these results. It would also be important to focus on exactly which XIST-related antigens contribute to female-biased immunity. Finally, the same authors also showed that patients with autoimmune diseases, including lupus, displayed significant autoantibodies to multiple components of XIST RNP, suggesting that the results in the mouse might be relevant for humans [101]. Nevertheless, autoantibodies against XIST binding proteins were also detected in male patients lacking XIST RNPs and, therefore, the importance of XIST RNPs for autoantibody production in lupus remains to be firmly validated.

Another study also suggests that the XIST lncRNA itself can be a female-specific self-antigen which is recognizable by TLR7 [102]. Using an unbiased search for highly expressed sex-biased transcripts with TLR7-stimulatory motifs, the authors pinpointed *XIST* as the strongest putative TLR7 ligand in female peripheral blood mononuclear cells (PBMCs). Transfection of specific XIST RNA motifs, including Repeat A, resulted in TLR7 stimulation and IFN-α production in pDCs. Conversely deletion of XIST in a cellular model decreased TLR7-mediated response. Interestingly, *XIST* levels exhibited elevation in PBMCs of women with lupus, particularly in B lymphocytes. These elevated levels demonstrated a positive correlation with both disease activity and the IFN signature. Additionally, XIST was found in extracellular vesicles released from cells undergoing in vitro cell death. These findings suggest that XIST is trafficked into extracellular vesicles during apoptosis, being exposed to TLR7 signaling, thus acting as an endogenous female-specific antigen with an immunostimulatory role in lupus [102]. Whether this occurs in vivo still needs to be analysed.

The notion of XIST and the proteins that form a complex with this long non-coding RNA as potential autoimmune targets (Fig. 4B) is relatively recent, necessitating further experimental evidence for validation, namely on actual biological material from patients. Nevertheless, it introduces an intriguing possibility where XIST itself and/or its protein partners could become targetable molecules for SLE treatment, both in women and men with Klinefelter syndrome.

### Increased escape from XCI

As mentioned earlier, XCI is not complete, with 15–25% of genes evading XCI in either a constitutive or facultative manner [87]. This results in women’s cells not only differing in terms of the parental X chromosome they have silenced but also in their ability to express specific X-linked genes from either one or both X chromosomes. Such diversity can be advantageous for women, allowing greater gene expression plasticity in certain contexts. However, inadequate fine-tuning of these mechanisms may leave them more susceptible to female-biased diseases. This was exactly the hypothesis proposed by several scientists that suggests that increased escape from XCI may play a role in female-biased autoimmune diseases, including lupus [103, 104].

While direct evidence linking enhanced XCI escape to lupus is currently lacking, a substantial body of data has been generated in the pursuit of such evidence. For instance, certain immune-related genes (e.g., *CD40L*, *CXCR3*, *BTK*, *IRAK-1*, *TLR7*, *TLR8*, *CXorf21*) have the capacity to partially evade XCI in healthy women, leading to biallelic expression in specific immune cell types (Table 1). Importantly, many of these genes are found to be overexpressed in lupus and other immune-mediated diseases. This observation suggests that an increase in the degree of escape could contribute to this overexpression. Escape from XCI has been comprehensively studied for *TLR7*, a gene that is hyperactivated in lupus and crucial for the production of IFN-α and other pro-inflammatory cytokines. In fact, through techniques measuring allelic expression of *TLR7* at single-cell resolution, researchers demonstrated that certain subsets of B lymphocytes, monocytes, and pDCs exhibit biallelic expression in women. These biallelic cells have elevated levels of *TLR7* mRNA compared to cells expressing *TLR7* from only one allele. Consequently, this leads to heightened TLR7 protein levels in PBMCs of women in comparison to men [105–107].

*TLR7* biallelic B lymphocytes are more likely than monoallelic cells to undergo immunoglobulin G class switch during B cell differentiation into immunoglobulin-secreting cells in response to TLR7 ligands in a T cell-dependent manner [105]. Conversely, pDCs in which *TLR7* evades XCI exhibit elevated levels of not only *TLR7* mRNA and protein, but also transcripts encoding IFN-α/β under steady-state conditions [106]. Hence, the allelic status of *TLR7* in B lymphocytes and pDCs alters immune cell function, influencing the overall immune response. Importantly, inter-individual variability has been noted with the frequency of biallelic *TLR7* cells in healthy women ranging from 5 to 40% in pDCs and 20–40% in B cells [105, 106]. Likewise, female monocytes in which *TLR8* is biallelically expressed, display higher abundance of TLR8 protein than male cells [108]. A tantalizing hypothesis would be that a higher prevalence of biallelic cells for *TLR7/8* or other immune-related X-linked genes will increase their protein levels and the susceptibility of women to develop lupus.

However, what could be the underlying mechanism driving an increased escape in XCI in lupus? Several studies have investigated the potential dysregulation of XIST in immune cells as a factor contributing to an aberrant active state of the Xi. Evidence of a role for XIST in XCI maintenance was established both in murine hematopoietic cells [109] and in human female B cell lines [78]. In the later study, targeted deletion and knockdown of *XIST* in a B lymphoblastoid cell line resulted in the unsilencing of several X-linked genes, including the immune-related *TLR7*, *CXCR3*, or *CXorf21*. Mechanistically, X-linked genes dependent on XIST-mediated silencing lack the DNA methylation at their promoters and regain histone acetylation upon *XIST* ablation. Furthermore, XIST silencing in primary B cells upon co-stimulation of B-cell receptor (BCR) and TLR7 resulted in the emergence of CD11c + atypical memory B cells, a cell population aberrantly expanded in autoimmune conditions, such as lupus [78]. Interestingly, a transcriptome signature indicating an increased escape of XIST-dependent genes was derived from single-cell RNA-seq data obtained from atypical memory B cells in female SLE individuals [78]. Additional validation is crucial to confirm these findings and grasp the mechanisms behind impaired XCI maintenance in atypical memory B cells and possibly other immune cell types in the context of the pathophysiology of lupus.

An additional research avenue has been centered on characterizing the pattern of *XIST* expression, predominantly employing RNA Fluorescent in situ hybridization (FISH), throughout hematopoiesis and immune activation in both healthy and diseased conditions. These studies showed that the expression pattern of *XIST* undergoes remarkable dynamic changes during hematopoiesis. Whereas hematopoietic stem cells, lymphoid and myeloid progenitors present a Xi territory covered by XIST (the characteristic XIST clouds) and the H3K27me3 chromatin mark, this is not the case in mature lymphoid cells and pDCs [110–112]. For example, during B and T cell development, the XIST compartment and the heterochromatin marks associated with the Xi are progressively lost [112]. Intriguingly, although the XIST compartment is no longer visible by RNA FISH in mature naive B and T cells, it is continuously transcribed and diffusely localized in the nucleus. This presents a potential opportunity for the reactivation of genes from the Xi (Fig. 4C), including, but not limited to, *TLR7*, *TLR8*, *CD40L*, *CRXC3* or *BTK.* However, it is important to note that conclusive evidence establishing a cause-and-effect relationship in this context is still lacking.

In vitro, upon activation of healthy mature B lymphocytes with LPS or CpG and T cells using CD3/CD28, XIST RNA and the repressive chromatin marks are relocated to the Xi [112]. Interestingly, such relocalization is impaired in B and T cells from individuals with lupus upon in vitro stimulation [13, 113] and phenocopied on the NZB/NZW F1 SLE mouse model [110]. In another SLE mouse model, NZM2328, this was also recapitulated on activated T cells and linked to female-biased upregulation of 32 X-linked genes, including the immune-relevant *Cfp*, *Foxp3*, *Cxcr3*, *Btk* and *Tlr8* [114]. However, the implications of this repositioning defect of *XIST/Xist* on the reactivation of Xi and consequent upregulation of these genes in activated SLE lymphocytes remain undefined, as comprehensive RNA FISH analysis of XIST clouds and escaping X-linked genes was not conducted.

Recently, two studies reported mouse models with decreased or no *Xist* expression [115, 116]. The first model involved a constitutional KO of the X-linked *Ftx* gene (*Ftx*-/-), a known activator of Xist [117], resulting in approximately half of the normal levels of *Xist* across different immune cell types [115]. The second model was a conditional KO of the Xist gene itself, using Cre recombinase under the *Mb1* promoter to delete the gene in the B cell lineage [116]. In both models, some female mice developed spontaneous systemic auto-immunity phenotypes with age, characterized by the production of SLE-specific autoantibodies and the expansion of certain immune cell subtypes, notably disease-relevant CD11c + age-associated B cells [115, 116] Splenomegaly was observed in the *Ftx*-/- model, and signs of glomerulonephritis were found in the B cell-specific *Xist* KO. These results link a reduction of *Xist* expression in one or more immune cell types and the development of SLE-like symptoms. Both models reported increased X-linked gene expression, although the effect was rather modest. In the *Ftx*-/- model, RT-qPCR revealed upregulation of a few genes, but without systematic RNAseq analysis, the full extent of this effect remains to be determined. For the X-linked genes, *Tlr7* and *CXorf21/Tasl*, this study showed a moderate increased reactivation in monocytes/macrophages and splenic B cells by RNA FISH. In the B cell-specific Xist KO model, only one X-linked gene, *CXorf21/Tasl*, was consistently upregulated across different B cell subtypes in both spontaneous and pristane-induced SLE models. However, direct evidence linking *CXorf21/Tasl* upregulation to Xi reactivation has not been formally tested. Future analyses will need to elucidate how much of the phenotypes could be caused by Xi reactivation. Additionally, it will be interesting to determine whether these effects are stronger in the more escape-prone human Xi, as suggested by in vitro studies using human lymphoblastoid cell lines [78]. Single-cell RNAseq of immune cells from patients and controls is necessary to determine if XIST expression fluctuates in SLE patients and to find any evidence of Xi reactivation in cells with low XIST expression.

In summary, conclusive evidence supporting the occurrence of increased Xi escape, potentially facilitated by XIST malfunction, in lupus is currently lacking. Regardless, the potential for increased XCI escape may be specific to certain immune cell types and specific circumstances during disease onset or progression. Unraveling the specifics of when, how, and in which cellular populations this phenomenon occurs will require further investigations.

## Perspectives and significance

Based on the evidence synthesized in this review, the association between the number of X chromosomes and lupus is robust, yet the underlying molecular mechanisms driving this association remain largely unresolved. As the process of XCI is intimately connected with the presence of two or more X chromosomes, extensive research has been directed towards elucidating whether the components involved in XCI or its dysregulation contribute to autoimmunity in general and lupus in particular. This review highlights three primary areas of investigation within this domain. The hypotheses outlined, including XCI skewing, XIST RNP complex as an autoantigenic target, and increased XCI escape, are not mutually exclusive and may collectively influence the pathogenesis of lupus. Some findings, however, particularly those from murine models with modulated *Xist* expression, might be challenging to reconcile, as both downregulation [115, 116] and upregulation [101] of Xist have been associated with SLE-like phenotypes. This discrepancy could be explained if XIST impacts autoimmunity and lupus differently depending on the disease stage or cell type. For example, a lack of XIST could promote dysregulated immune responses within certain immune cell types, such as B cells and pDCs, while the XIST RNP complex might enhance autoimmunity in damaged tissues. It is important to recognize that the translatability of these results to the human context may be limited. SLE-like mouse models do not accurately represent the long-term progression, disease presentation, and chronicity of human SLE as previously mentioned. Additionally, significant differences in XCI regulation and the number of XCI escapees between species further complicate the reliance on mouse models for studying lupus. For example, relatively modest alterations in X-linked gene expression have been observed in the B cell compartment of mice [116], and it remains unclear whether major changes would occur on a more escape-prone human Xi in vivo. Evaluating whether increased XCI escape or elevated autoantibodies against XIST-binding proteins occur during disease onset or flares in humans would be critical to ascertain the significance of these processes in disease onset and progression. However, conducting such experiments in typical clinical settings would present substantial challenges and could be misleading if not correctly controlled. In any case, continued exploration into the intricate role of the X chromosome and the process of XCI in lupus will enhance our understanding of the strong sex bias observed in this disease. Moreover, such investigations may uncover a plethora of novel target genes, whether they involve XIST and its protein partners or other X-linked genes, that, ultimately, could, hopefully in the long run, pave the way for a better understanding of one of the most prevalent autoimmune diseases affecting girls and women.

## Data Availability

Not applicable.
